# Epithelial Ingrowth Following Pterygium Excision With Conjunctival Autograft: A Rare Complication

**DOI:** 10.7759/cureus.82416

**Published:** 2025-04-17

**Authors:** Loubna Mouhib, Othman Haddani, Mehdi Khamaily, Mohamed Elbelhadji, Abdelbarre Oubaaz

**Affiliations:** 1 Ophthalmology, Mohammed VI University of Health Sciences (UM6SS), Casablanca, MAR

**Keywords:** complication of pterygium surgery, epithelial cells, epithelial ingrowth, ocular surgeries, pterygium surgery

## Abstract

Pterygium is a common ocular condition characterized by a conjunctival growth over the cornea. Epithelial ingrowth (EI), a rare complication of ocular surgery, involves the proliferation of epithelial cells within the cornea or the anterior chamber. We report a case of EI in a male patient following pterygium surgery with conjunctival autograft. Slit lamp examination revealed a thin, grey-white, wave-like band beneath the corneal epithelium in the inferior-temporal quadrant, outside the visual axis and not crossing the corneal center. The diagnosis of epithelial ingrowth was made clinically and confirmed with anterior optical coherence tomography (OCT). This marks only the second documented occurrence of EI after pterygium surgery.

## Introduction

Epithelial ingrowth (EI) is an abnormal epithelial membrane that develops within the eye; it can spread over the posterior surface of the cornea and infiltrate the anterior chamber angle [1], inside the cornea between the stroma and Bowman membrane or under a LASIK flap [2]. It can lead to significant clinical manifestations such as glaucoma or corneal edema [1]. This condition is a known complication of incisional ocular procedures and LASIK [2]. At the time of writing, only one prior case of epithelial ingrowth following pterygium surgery has been identified in the literature [1], marking this as the second documented occurrence.

## Case presentation

**Figure 1:**
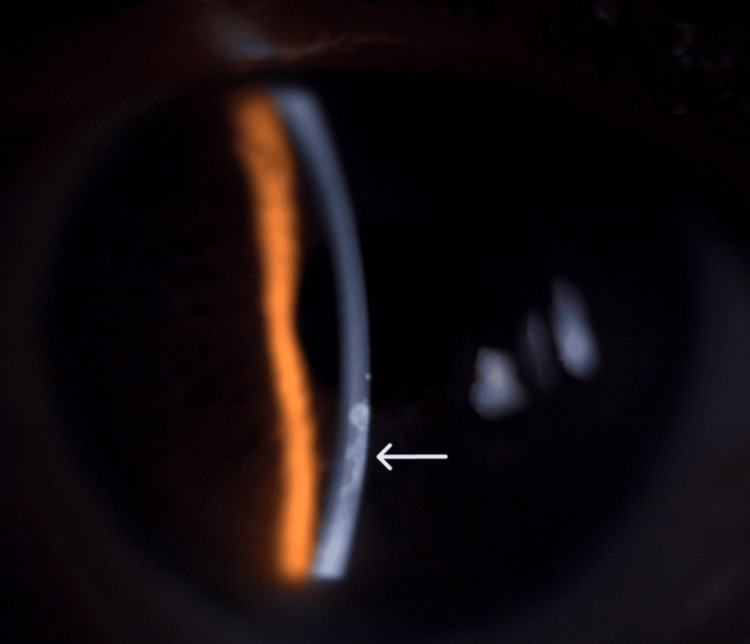
Epithelial ingrowth beneath the corneal epithelium on slit lamp with a 30° view (white arrow)

**Figure 2:**
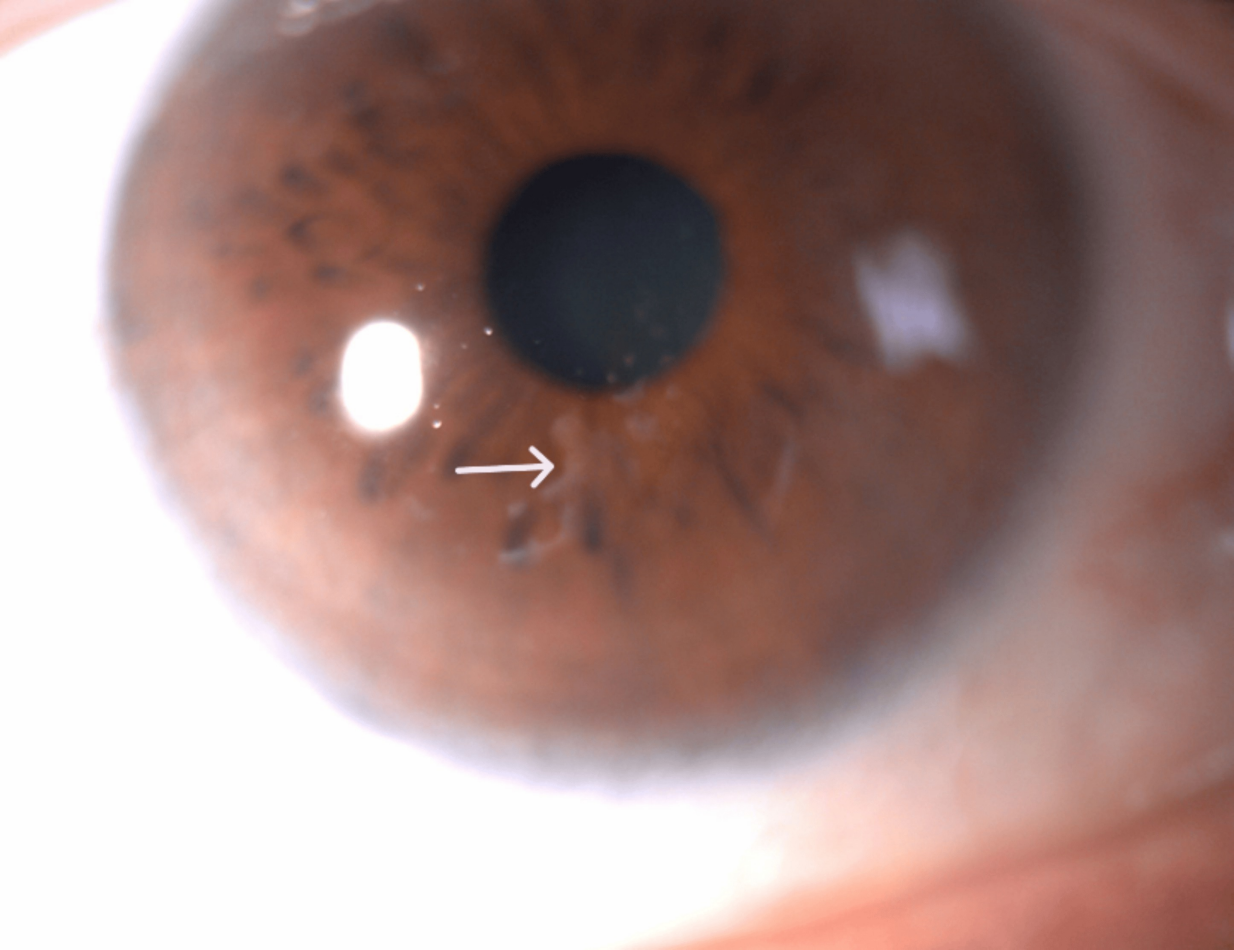
Epithelial ingrowth on the inferior-temporal quadrant of the cornea (white arrow)

A 63-year-old male attended an ophthalmic consultation due to monocular diplopia and blurred vision in his right eye. He had undergone temporal pterygium excision surgery with conjunctival autograft in the right eye eight weeks earlier with no perioperative complications. His best-corrected distance visual acuity was 20/22 OD (+1.50 (-1.50 at 115°)) and 20/20 OS (+1.50 (-1.50 at 115°)) with intraocular pressure of 18 and 19 mmHg, on-air tonometry confirmed by applanation. On slit-lamp biomicroscopic examination, a thin, grey-white band beneath the corneal epithelium with a wave-like shape was observed in the inferior-temporal quadrant of the cornea (Figure 1). The lesion was outside the visual axis and wasn’t crossing the center of the cornea (Figure 2).

**Figure 3:**
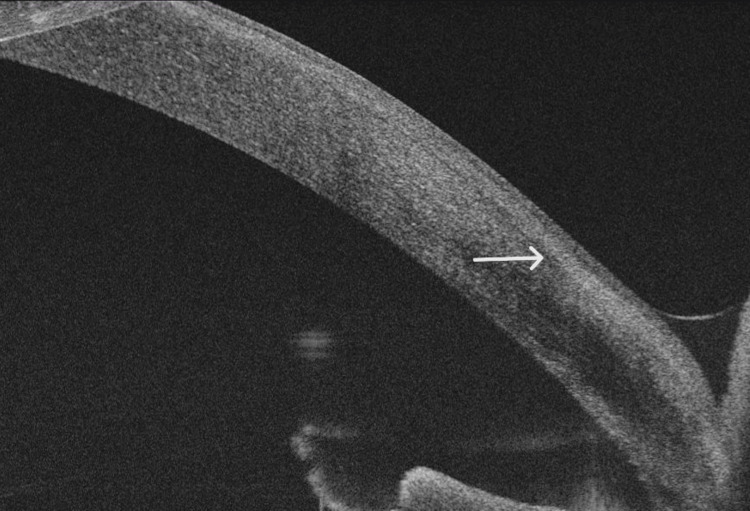
Anterior optical coherence tomography (OCT) showing hyper-reflective membrane between the stroma and the corneal epithelium (white arrow)

The endothelium and anterior chamber structures were unremarkable. On gonioscopy, there were no epithelial cells in the angle. The fundus examination revealed no abnormalities in either eye. Anterior segment optical coherence tomography (OCT) showed multiple discrete hyper-reflective membranes beneath the corneal epithelium and the Bowman membrane inside the anterior stroma (Figure 3). 

The anterior OCT and clinical examination confirmed the diagnosis of epithelial ingrowth. Various methods can be used to treat epithelial ingrowth. We recommended therapeutic photo keratectomy (TPK), but the patient declined because he was satisfied with his new optical correction and aware of the high recurrence rate associated with the treatment. 

## Discussion

**Figure 4:**
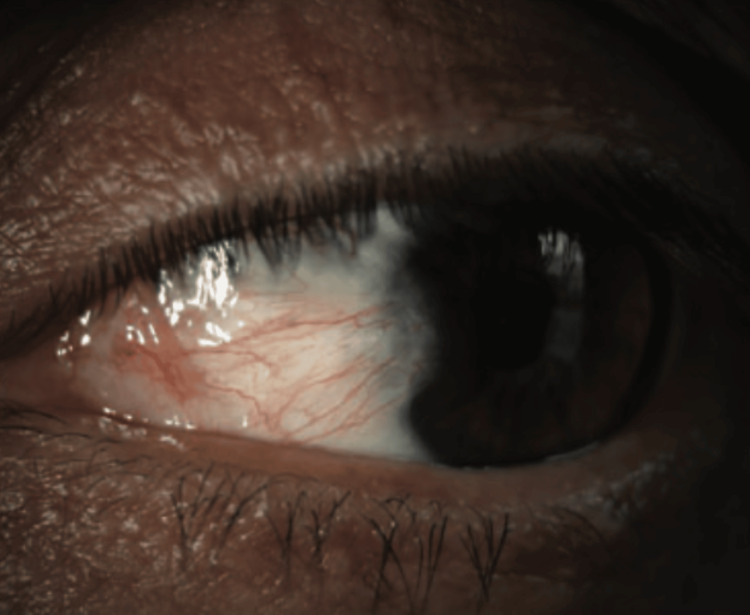
Temporal pterygium with a corneal invasion Reproduced under CC BY-NC 4.0 from [3]

**Figure 5:**
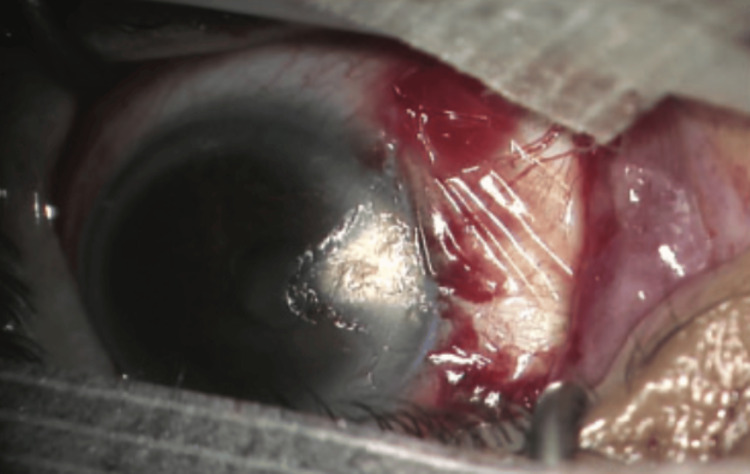
Pterygium removal with amniotic membrane graft Reproduced under CC BY-NC 4.0 from [3]

Pterygium is a common ocular condition characterized by conjunctival growth over the cornea (Figure 4). It can develop on either the nasal or temporal side, potentially affecting the cornea and causing astigmatism. Several surgical techniques are available for the pterygium. After it is removed, it can be replaced by a conjunctival autograft, conjunctival suture, or amniotic membrane (Figure 5) [3].

The conjunctival autograft is the most commonly used method, despite its reported recurrence rate of 31.3% [4]. Surgical complications associated with these procedures are uncommon. Rare instances of complications in pterygium treatment include scleral ulceration, corneal infection, graft rejection, poor epithelial healing, glaucoma, endophthalmitis, and superficial punctate keratitis have been reported [5]. Epithelial ingrowth is an infrequent complication following various types of ocular surgeries. Our case marks the second documented instance after pterygium surgery [1].

After surgery on the cornea, epithelial cells begin their healing process by releasing many cytokines such as interleukin-1, epidermal growth factor, and tumor necrosis factor-alpha (TNF-a) [6]. This healing process is the first step of the pathogenesis of the epithelial ingrowth [7]. Epithelial conjunctival cells then disseminate inside a space (intra-stromal or intraocular). These epithelial cells can multiply due to the cytokine storm inside a tissue without destroying it. It results in a membrane on the endothelium that can spread to the iridocorneal angle or inside a space in the cornea (Lasik flap, pterygium scar).

The hypothesized mechanism in our case was likely an overly deep excision of the pterygium at the corneal level, reaching the anterior stroma, where epithelial cells adhered and proliferated on this newly created space. This condition can cause decreased vision by secondary astigmatism or by affecting the visual axis [8]. It is not the case with our patient.

Khurana et al. reported a case of epithelial ingrowth following cataract surgery and recommended avoiding incisions at the pterygium site [8]. Besides cataract and pterygium surgery, epithelial ingrowth is a well-known complication in the refractive surgery field [2]. There are reports of post-Lasik epithelium ingrowth (PLEI) [2] in the early postoperative period. The incidence is between 0-3.9% [9] for the first LASIK treatment, and more around 10-20% in cases of re-treatment [10].

For diagnostic purposes, confocal microscopy can help confirm that the epithelial cells exhibit characteristics consistent with conjunctival cells [8].

EI treatment methods vary based on the location of the epithelial cells. If the cells invade the iridocorneal angle and give rebel hypertonia to medical treatment, a trabeculectomy with mitomycin C should be performed to help lower the hypertonia [1]. When the EI invades a significant portion of the corneal endothelium resulting in various symptoms, a penetrating keratoplasty [1] or a Descemet membrane endothelial keratoplasty (DMEK) can be performed. If the cells develop on the anterior layers such as the anterior stroma or Bowman membrane, less invasive techniques can be used, such as anterior lamellar keratoplasty (ALK) or therapeutic photokeratectomy (TPK) [10]. 

Monitoring the patient is recommended if the EI doesn’t reach the visual axis and doesn’t have significant symptoms, like in our case. In PLEI, it is suggested that the best method is the flap opening with mechanical debridement of the cells [11]. Closing the flap with a 10-0 suture or fibrin glue may reduce the recurrence risk [2]. Another technique for the PLEI that has been reported and tested involves noninvasive neodymium-doped yttrium aluminum garnet (Nd:YAG) [12], with low energy in an average of 0.6 MJ. Various other methods appear in the literature including approaches such as wide excision with a corneoscleral patch graft, transcorneal cryotherapy, 5-fluorouracil, or mitomycin-C [13-15]. With all these therapeutic options, it is advised to keep in mind that EI has a high rate of recurrence after treatment [8].

## Conclusions

Epithelial ingrowth is an infrequent complication following pterygium surgery. It has been described in other conditions such as post-LASIK or incisional surgeries. Therapeutical options are non-codified, and should be evaluated on an individual basis, taking into account the symptoms, clinical characteristics, and cell location.
